# The histone deacetylase inhibitor, sodium butyrate, exhibits neuroprotective effects for ischemic stroke in middle-aged female rats

**DOI:** 10.1186/s12974-016-0765-6

**Published:** 2016-12-01

**Authors:** Min Jung Park, Farida Sohrabji

**Affiliations:** Department of Neuroscience and Experimental Therapeutics, Women’s Health in Neuroscience Program, College of Medicine, Texas A&M University Health Science Center, 8447 State Highway 47, Bryan, TX 77807 USA

**Keywords:** Ischemic stroke, Inflammation, Oxidative stress, Histone deacetylase inhibitor, Sodium butyrate, Insulin-like growth factor-1, Cytokines, Middle-aged female

## Abstract

**Background:**

Sodium butyrate (NaB) is a histone deacetylase (HDAC) inhibitor exhibiting anti-inflammatory and neuroprotective effects in a rat ischemic model of stroke as well as a myocardial ischemia model. Although clinical evidence shows that older women are at higher risk for stroke occurrence and greater stroke severity, no studies have evaluated the effectiveness of NaB either in females or in older animals.

**Methods:**

To determine the effects of NaB on stroke in older females, acyclic middle-aged Sprague-Dawley female rats (9–11 months old, constant diestrus) were subject to middle cerebral artery occlusion (MCAo) by intracerebral injection of recombinant endothelin-1. Rats were treated with NaB (300 mg/kg, i.p.) at 6 and 30 h following ET-1 injection. Animals were sacrificed at the early (2 days) or late (5 days) acute phase after MCAo. Serum and tissue lysates were collected for biochemical analyses.

**Results:**

NaB treatment reduced infarct volume and ameliorated sensory motor impairment in middle-aged female rats, when measured at 2 and 5 days post MCAo. At the early acute phase (2 days post stroke), NaB treatment decreased brain lipid peroxides, and reduced serum levels of GFAP, a surrogate marker of blood-brain barrier (BBB) permeability. NaB also reduced expression of the inflammatory cytokine IL-1beta in circulation and IL-18 in the ischemic hemisphere. At the late acute phase (5 days post stroke), NaB treatment further suppressed MCAo-induced increase of IL-1beta, IL-17A, and IL-18 in brain lysates (cortex and striatum) from the ischemic hemisphere, and decreased ischemia-induced upregulation of IL-1beta and IL-18 in circulation, indicating a potent anti-inflammatory effect of the HDAC inhibitor. Moreover, NaB treatment also increased expression of IGF-1, a known neuroprotectant, in peripheral tissue including serum, liver, and spleen at the late acute phase.

**Conclusions:**

These data provide the first evidence that delayed (>6 h) NaB treatment post-stroke is neuroprotective in older female rats. Additionally, these data also show that in addition to its well-known anti-inflammatory actions, NaB may exert a biphasic effect after stroke, operating initially to reduce BBB permeability and oxidative stress in the brain, and later, elevating IGF-1 expression in peripheral tissues.

## Background

Stroke is the fifth-leading cause of death in the USA and the most common cause of disability [1]. Ischemic stroke is more prevalent in the elderly, and among this population, postmenopausal women have a higher risk for stroke occurrence, greater stroke severity, and slower recovery [2, 3]. Recent preclinical studies have accurately replicated the demographic differences in which aging female mice present increased stroke damage as compared to aging male mice [4]; and acyclic middle-aged female rats show greater infarct volume and behavioral deficit as compared to young female rats [5].

Histone acetylation and deacetylation are the two major players in epigenetic mechanisms to regulate transcription and other functions in cells including neurons, microglia, and astrocytes. Protein acetylation, catalyzed by histone acetyltransferases (HATs), at histone proteins results in an open chromatin conformation, thus stimulating transcription and activating gene expression. The other modifier, histone deacetylases (HDACs), catalyzes deacetylation of histone proteins at lysine (Lys, K) residues, hence inhibiting transcription and gene expression. The levels of histone acetylation (thereby remodeling chromatin structure) is determined by the balance between HATs and HDACs. In general, HDACs function as a transcriptional repressor to silence gene expression and induce chromatin compaction. Therefore, HDAC inhibition alters the balance towards enhancing histone acetylation, chromatin relaxation and gene expression [6, 7]. Histone hypoacetylation and chromatin compaction have been reported in rodent studies of middle cerebral artery occlusion (MCAo). A fatty acid derived HDAC inhibitor, sodium butyrate (NaB) blocks class I and IIa HDACs and is known to readily cross the BBB [8]. NaB exhibits anti-inflammatory and neuroprotective effects in a rat ischemia model of stroke as well as myocardial infarction through multiple mechanisms including reducing infarct volume, enhancing neurogenesis, and reducing pro-inflammatory cytokines in the ischemic brain [9–14]. Administration of NaB immediately after MCAo (and at various time points) presents neurogenic effects mediated by the BDNF-TrkB signaling pathways and anti-inflammatory effects by blocking inducible nitric oxide and COX-2 induction in a male rodent model of ischemia [11, 13].

Unfortunately, no studies have evaluated the effectiveness of the HDAC inhibitors in animal models that approximate stroke prone groups such as elderly females. Older women are more likely to suffer a stroke and have worse stroke impairment, thus the present study tested the effectiveness of delayed NaB administration in middle-aged female rats during the early and late acute phase after cerebral ischemia. The early acute phase of stroke spans minutes to hours (24–48 h) after ischemia, and is marked by excitotoxicity including increased reactive oxygen species, glutamate release, with activation of local inflammatory cells and rapid necrotic cell death of neurons [15, 16]. During the late acute phase, inflammation persists and apoptotic cell death is observed as well as repair and regeneration such as neurogenesis, angiogenesis and sprouting [17, 18]. Our data shows that NaB treatment to middle age female rats after stroke modifies key features of the acute phase of ischemic stroke.

## Methods

### Ethics statement

All experimental protocols were approved by the Texas A&M University Institutional Animal Care and Use Committee. All animal care and use was conducted in accordance with the Guide for the Care and Use of Laboratory Animals (National Research Council).

### Animals and estrous cycle determination

Female Sprague-Dawley rats (*n* = 75) were purchased from Envigo (Houston, TX) as middle-aged animals (9–11 months, 280–360 g). All animals were maintained in temperature (22 °C) and humidity (45–55%) controlled environment with a 12/12 h dark-light cycle (0700 to 1900 h). Rats were fed pelleted food (Harlan, Teklad Rodent Diet) and water ad libitum. One week after arrival, animals were subject to daily vaginal smears to determine estrous status as reported previously [19]. Briefly, collected vaginal cells were placed on slides and cell cytology was examined. Middle-aged female rats were selected if cell cytology indicated they were in constant diestrus for at least seven consecutive days. To confirm estrus status, serum samples were collected and measured for estradiol (Cayman Chemical, MI). Serum estradiol levels in this group were below the detection limit (< 6.6 pg/ml) of the assay.

### Middle cerebral artery occlusion (MCAo) and sodium butyrate treatment

MCAo was induced by intracerebral injection of endothelin-1 (ET-1) to the MCA as previously described [20]. Animals were anesthetized (100 mg/ml/kg ketamine and 20 mg/ml/kg xylazine) and placed in a stereotaxic apparatus. ET-1 (3 microliters of 0.5 μg/μl, 600 pmol) was injected at a rate of 1 microliter per minute to the left middle cerebral artery (AP: +0.9, ML: −3.4, relative to bregma, DV: −8.5, relative to dura). After the surgery, rats were treated with NaB (Sigma-Aldrich, MO, 300 mg/kg, i.p.) at 6 and 30 h following ET-1 injection.

### Infarct volume

Animals were euthanized at the early (2 days) or late (5 days) acute phase after MCAo. Brains were quickly removed and sliced coronally at 2-mm thickness. The sections were immersed in 2% 2,3,5-triphenyltetrazolium chloride (TTC) in DPBS for 30 min at 37 °C and processed for imaging. The digitized images were used to quantify infarct volume using the Quantity One software package (Bio-Rad, CA). Infarct volume estimation was performed as reported previously [5]. Briefly, the posterior (caudal) face of each section from three consecutive sections was analyzed in each animal. The infarct area of two adjacent slices was averaged and then multiplied by the thickness of the slice, and values across all slices were added to obtain the volume of the infarct. The volume of the ischemic zone and the total volume of non-ischemic hemisphere was measured separately, and infarct volume is reported as the ratio of the ischemic volume to the non-ischemic hemisphere.

### Behavioral assays

Motor impairment following MCAo was assessed using the vibrissae-evoked forelimb placement task [5] and the adhesive-tape test [21] as described previously. The vibrissae-elicited forelimb placement test was used 3 and 2 days before and 2 and 5 days after the MCAo surgery. Animals were subject to same-side placing trials and cross-midline placing trials elicited by brushing the ipsi- and contra-lesional vibrissae against the edge of a table. During the same-side forelimb placing trials, the animal’s ipsilesional vibrissae were stimulated against the edge of a table and forelimb placing response on that side was scored by an investigator, who was blinded to experimental conditions. In the cross-midline placing trials, the animal was held gently by the upper body such that the ipsilesional vibrissae lie perpendicular to the table top and the forelimb on that side is gently restrained as the vibrissae was brushed on the top of the table to evoke a response from the contralesional limb and vice versa. Between each trial the animal was allowed to rest all four limbs briefly on the table top to help relax its muscles. Ten trials were performed during each test.

The adhesive tape test was performed 2 days before and 2 or 5 days after surgery. Two pieces of adhesive-backed foam tape (Scotch Permanent Mounting Squares, 12.7 × 12.7 mm) were used as bilateral tactile stimuli attached to the palmar surface of the paw of each forelimb. For each forelimb, the time it took to remove each stimulus (tape) from the forelimbs was recorded during three trials per day for each forepaw. Animals were allowed to rest for 1 min between sessions, and each test session had a maximum time limit of 120 s.

### Protein extraction and quantification

Cortex (parietal and temporal) and striatum from the ischemic and non-ischemic hemisphere, liver, and spleen samples were collected and homogenized in lysis buffer (50 mM Tris, pH 7.4, 150 mm NaCl, 10% glycerol, 1 mM EGTA, 1 mM Na-orthovanadate, pH 10, 5 μM ZnCl2, 100 mM NaF, 10 μg/ml aprotinin, 1 μg/ml leupeptin, 1 mM phenylmethylsulfonyl fluoride in dimethylsulfoxide, 1% Triton X-100), and lysates were collected after centrifugation at 20,000*g* for 30 min. Protein concentrations were determined using the BCA protein assay kit (Pierce, IL) and the plates were read at 562 nm in a microplate reader (Tecan Infinite® 200 PRO).

### Cytokine/chemokine measurements

Expression levels of a panel of inflammatory cytokine/chemokine were quantified using a rat cytokine/chemokine panel (Millipore, MA). The procedure was performed according to the manufacturer's directions. Samples, standards, and controls were added to appropriate wells in a 96-well plate as stated in the kit protocol and incubated with premixed beads at room temperature for 2 h on a horizontal orbital microplate shaker. After washes (2X), 25 μL of detection antibodies was added to each well, incubated at room temperature for 1 h on the shaker, and 25 μL of streptavidin-phycoerythrin was added to each well containing the 25 μL of detection antibodies. After 30 min of incubation at room temperature, the wells were washed (2X) and filled with sheath fluid. The plate was read on a Bio-Plex System (Bio-Rad, CA). Brain cytokines and chemokine levels were normalized to total protein concentrations.

### Measurement of serum and tissue IGF-1 levels

IGF-1 levels were measured using a commercial solid phase sandwich rat ELISA kit (R&D systems, MN) as per manufacturer's instruction. Samples, standards, and controls were added to appropriate wells in a 96 well plate as stated in the kit protocol and incubated at room temperature for 2 h on a horizontal orbital microplate shaker. After wash, 100 μL of conjugate was added to each well and incubated at room temperature for 2 h on the shaker. After wash and incubation in substrate solution for 30 min, the plates were read at 450 nm in a plate reader with wavelength correction to 540 nm (Tecan US Inc., Durham, NC). Sample measurements were interpolated from the standard curve, and values from tissue lysates were normalized to total protein concentrations.

### Measurement of serum and tissue IGFBP-3 levels

IGFBP-3 levels were measured using a commercial ELISA kit (Crystal Chem, IL) as per manufacturer's instruction. Samples, standards, and controls were added to appropriate wells in a 96 well plate as stated in the kit protocol and incubated at room temperature for 1 h on a horizontal orbital microplate shaker. After washes (5X), 100 μL of antibody conjugate was added to each well and incubated at room temperature for 1 h on the shaker. After washes (5X), 100 μL of enzyme conjugate was added to each well and incubated at room temperature for 15 min on the shaker. After wash and incubation in 100 μL of substrate solution for 15 min in a dark room, 100 μL of stop solution was added and the plates were read at 450 nm in a plate reader with wavelength correction to 630 nm (Tecan US Inc., Durham, NC). Sample measurements were interpolated from the standard curve, and values from tissue lysates were normalized to total protein concentrations.

### Measurement of serum GFAP levels

Glial fibrillary acidic protein (GFAP) levels were measured using a commercial ELISA kit (Millipore, MA) as per manufacturer’s instruction. Samples, standards, and controls were added to appropriate wells in a 96 well plate as stated in the kit protocol and incubated at room temperature for 2 h on a horizontal orbital microplate shaker. After washes (4X), 100 μL of biotinylated anti-GFAP detection antibody was added to each well and incubated at room temperature for 1 h on the shaker. After wash, 100 μL of enzyme solution was added to each well and incubated at room temperature for 30 min on the shaker. After washes (6X) and incubation in 100 μL of substrate solution for 15 min in dark room, 100 μL of stop solution was added and the plates were read at 450 nm in a plate reader (Tecan US Inc., Durham, NC). Sample measurements were interpolated from the standard curve.

### Thiobarbituric acid reactive substances (TBARS) assay

Lipid peroxidation in the ischemic and non-ischemic hemispheres was determined using the TBARS assay kit (Cayman Chemical, MI) according to manufacturer’s instructions and as described previously [22]. Briefly, a mixture of 25 μl of sample, standard and 25 μl of SDS was prepared. To this mixture, 1 ml of color reagent was added and boiled for an hour in boiling water bath. The reaction was stopped on ice by 10 min incubation and centrifuged for 10 min at 1600*g* at 4 °C. The supernatant (150 μl) was loaded on a 96-well plate and absorbance was read at 540 nm in Tecan plate reader. TBARS concentration was calculated from a malondialdehyde standard curve using Magellan software and normalized to the amount of total protein.

### Statistical analysis

Power analysis, using data from pilot studies and other experiments using middle aged females, estimated sample size at five. Most groups had a larger sample size, with five as the minimum. For infarct volume, a two-way ANOVA coded for treatment and day was used. For behavioral tests, a paired Student’s *t* test was used for each group, comparing the values obtained pre- and post-stroke. For all other comparisons an unpaired Student’s *t*-test or a two-way ANOVA was used. Group differences were considered significant at *p* < 0.05 in each case. All data presented in bar graphs are the mean ± S.E.M. from multiple determinations. Specific animal numbers used for an assay is described in each figure legend.

## Results

### Sodium butyrate reduces post-stroke brain infarct volume

**Fig. 1:**
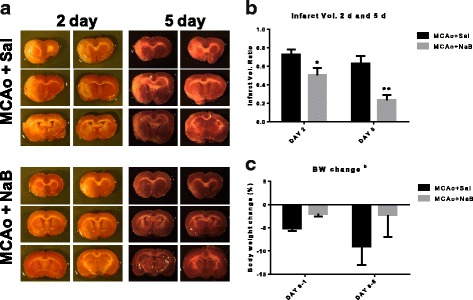
Sodium butyrate reduces brain infarct volume. Middle-aged female rats were subjected to MCAo and injected with two doses of vehicle or sodium butyrate each at 6 and 30 h after ET-1 injection. **a** Representative pictures of TTC-stained coronal sections from vehicle (saline)- or NaB (300 mg/kg, i.p.)-treated female rats at 2 and 5 days post stroke. **b** Quantitative analysis of infarct volume, expressed as a ratio to the non-ischemic hemisphere, shows that post-stroke NaB treatment significantly decreased infarct size compared to control at 2 and 5 days post stroke. **c** Percent body weight change during 24 h and 5 days post stroke, normalized to pre-stroke weight, indicates percent weight loss post MCAo is significantly reduced in NaB-treated animals as compared to control group during 24 h after MCAo. Graphs represent mean ± S.E.M, *n*  =  6–7 in each group. Unpaired *t* test (**b**) and two-way ANOVA, Tukey’s multiple comparisons test (**c**), **p < 0.01, *p < 0.05. b: main effect of treatment, *p* < 0.05

Sodium butyrate (NaB), a short chain fatty acid that blocks class I and IIa HDACs [7], was examined for its neuroprotective effects following stroke in middle-aged female rats. Twelve-month-old female rats were subjected to MCAo by ET-1 and injected with vehicle or NaB (300 mg/kg, i.p.) at 6 h after ET-1 injection, followed by another injection at 30 h after stroke onset. Figure 1a shows representative TTC-stained coronal sections of two animals from each group and Fig. 1b presents the quantification of the infarct volume normalized to the non-ischemic hemisphere. As shown in Fig. 1a, all of the groups showed cortical and striatal infarction, however, the extent of cell death was significantly affected by treatment [F _(1, 21)_, 19.93, *p* = 0.0002] and day [F _(1, 21)_, 6.96, *p* = 0.0154] (Fig. 1b). Post-stroke NaB treatment significantly decreased brain infarction at 2d (T-test, *p* = 0.0476, *n* = 6/group), and 5d (*t* test, *p* = 0.0018, *n* = 6–7/group) (Fig. 1b). The neuroprotective effect of post-stroke NaB at resulted in a 30% decrease in infarct volume at day 2 and a 63% decrease at day 5 as compared to post-stroke saline group (Fig. 1b).

Percent body weight change, as an indicator of sickness and safety of NaB treatment, was also measured during 24 h and 5 days post stroke (Fig. 1c). The extent of body weight change was significantly affected by treatment [F _(4, 46)_, 4.46, *p* = 0.0409] (Fig. 1c). Relative body weight change showed that the percent weight loss post MCAo was significantly reduced in NaB-treated animals (−2.013 ± 0.52%, *n* = 13) compared to control (−5.112 ± 0.63%, *n* = 14) during 24 h after MCAo (Fig. 1c).

### Sodium butyrate treatment ameliorates stroke-induced loss of sensory motor function

MCAo results in motor and sensory deficit in patients and animal models [23, 24]. To measure the extent of motor impairment following MCAo, two tests were utilized.

**Fig. 2:**
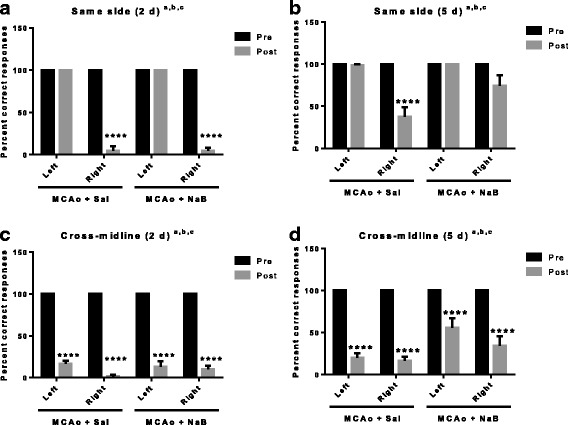
Post stroke sodium butyrate treatment improves percent correct responses in the vibrissae-elicited forelimb placement test. **a** The same side test of the vibrissae-elicited forelimb placement task at 2 days shows both post-stroke saline- (MCAo + Sal) and NaB-treated (MCAo + NaB) animals display significantly reduced percent correct responses in the contralesional paw placement as compared to pre-stroke, but not in the ipsilesional side. **b** At 5 days post stroke, control treated animals showed significant deficits in paw-placement on the contralesional side compared to the pre-stroke test. NaB-treated animals showed no significant difference in contralesinal paw-placement pre and post stroke. No pre and post stroke differences were seen on the ipsilesional side. **c**, **d** In the cross-midline test of the vibrissae-elicited forelimb placement task, all groups showed reduction in correct responses at 2 days (**c**) and 5 days (**d**). (All *graphs* represent mean ± S.E.M. *n* = 7–9 in each group; ****p < 0.0001; paired *t* test between pre and post. a: main effect of time (pre and post), *p* < 0.05; b: main effect of treatment, *p* < 0.05; c: main effect of interaction, *p* < 0.05

Vibrissae-evoked forelimb placement task: In the ‘same side’ test at 2 days post stroke, both post-stroke saline- and NaB-treated rats displayed significantly lower percent correct responses in the contralesional paw placement (*p* < 0.0001, *n* = 6), but not on the ipsilesional side (*p* > 0.9999, *n* = 6) (Fig. 2a). In contrast to 2 days, 5 days post-stroke saline-treated rats displayed significantly lower percent correct responses in the contralesional paw placement (*p* < 0.0001, *n* = 7–9), but not on the ipsilesional side (*p* > 0.9999, *n* = 7–9) (Fig. 2b). Importantly, no significant alterations were observed in post-stroke NaB-treated rats both in the contralesional (*p* = 0.0880, *n* = 7–9) and ipsilesional (*p* > 0.9999, *n* = 7–9) paw placement (Fig. 2b), indicating improved sensory motor function in post-stroke NaB group as compared to post-stroke saline treated animals at 5 days. This result is consistent with the decreased infarct volume in the post-stroke NaB group at 5 days (Fig. 1b). In the cross-midline test, both post-stroke saline- and NaB-treated animals showed significant post-stroke sensory motor deficit in the contralesional and ipsilesional paw placement at 2 and 5 days (Fig. 2c, d).

**Fig. 3:**
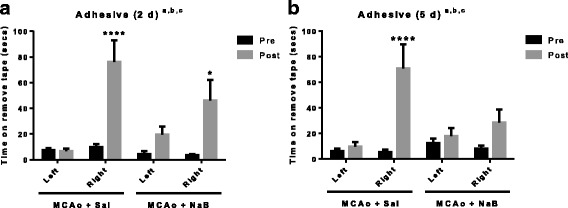
Sodium butyrate treatment improves the latency of tape removal post ischemia. **a** The latency (in seconds) to tape removal from the forepaw was evaluated before (pre) or after (2 days post) MCAo. Post-stroke performance on the ipsilesional paw in both saline- and NaB-treated animals was not significantly impaired at 2 days. However, post stroke performance on the contralesional paw was significantly worse in both groups. Latency to adhesive removal was significantly lower in the NaB treated group as compared to saline controls. **b** At 5 days post MCAo, post-stroke performance on the ipsilesional paw was not affected in either the saline- or NaB-treated animals. On the contralesional paw, post-stroke performance was significantly impaired in saline-treated animals, while no significant differences were found in post-stroke NaB-treated group. All graphs represent mean ± S.E.M. *n* = 7–9 in each group; *p < 0.05; paired *t* test between pre and post. **a** main effect of time (pre and post), *p* < 0.05; **b** main effect of treatment, *p* < 0.05; **c** main effect of interaction, *p* < 0.05

Sensory motor performance was also assessed by the latency to remove an adhesive pad from the forepaw (adhesive tape test). Post-stroke performance on contralesional paw was significantly impaired in both post-stroke saline and post-stroke NaB-treated animals at 2d (Fig. 3a, *p* < 0.0001 and *p* = 0.0208, respectively). Notably, the latency to tape removal from post-stroke NaB treated group (31.56 ± 6.93 s) was significantly reduced as compared to post-stroke saline treated group (76.08 ± 16.87 s, *p* = 0.0499, *t* test), indicating better sensory motor function in post-stroke NaB-treated animals at the early acute phase (2 days post stroke). At 5 days post stroke, the latency to tape removal on contralesional paw was significantly reduced in saline-treated group as compared to pre-stroke latency (*p* < 0.0001) while no significant difference was found in pre- and post-stroke latency of NaB-treated animals (*p* = 0.8083), indicating significantly better sensory motor function in post-stroke NaB-treated animals (Fig. 3b).

Combined, these data indicate that NaB attenuates MCAo-induced sensory motor deficit in middle-aged female rats (Figs. 2 and 3).

### NaB reduces blood brain barrier permeability in the early acute phase

**Fig. 4:**
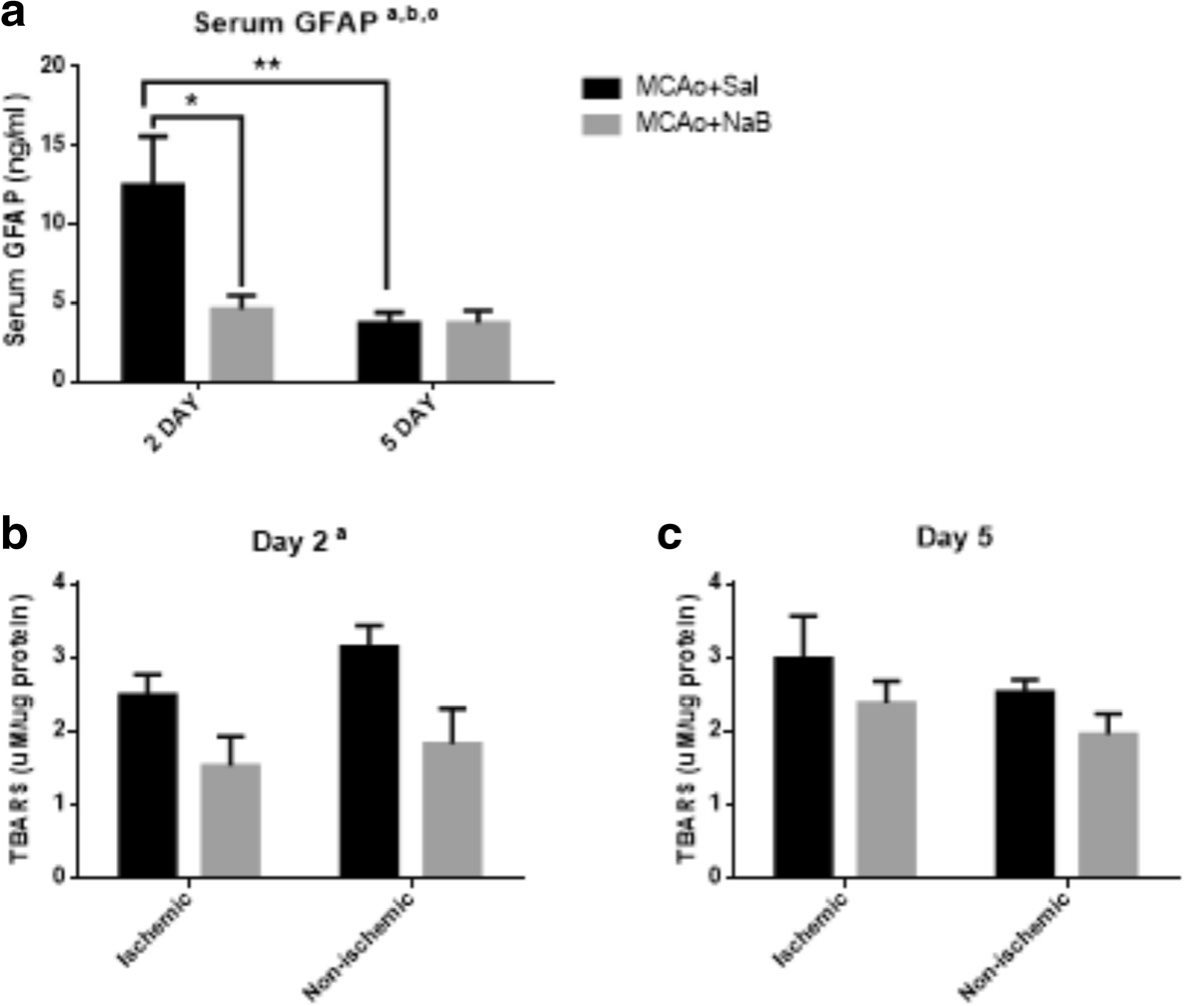
NaB treatment attenuates stroke-induced serum GFAP and brain TBARS at 2 days post stroke. **a** GFAP levels were measured from serum samples at 2 and 5 days post stroke. (B-C) TBARS levels were assessed from brain lysates at 2d (**b**) and 5d post stroke (**c**). All graphs represent mean ± S.E.M. *n* = 3–6 in each group. *p < 0.05 a: main effect of treatment, b: main effect of time, **c**: interaction effect (treatment X time). Two-way ANOVA with Tukey’s post hoc test. Repeated measures analysis (ischemic and non-ischemic hemisphere) for TBARS data

HDAC inhibitors are reported to augment blood brain barrier integrity [10]. We assessed blood-brain barrier permeability by measuring serum levels of glial fibrillary acidic protein (GFAP), which is a well-known biomarker of brain injuries including stroke and trauma [25–27]. Post-stroke saline-treated animals displayed a threefold higher serum level of GFAP (12.52 ± 3.11 ng/ml) as compared to post-stroke NaB-treated group (4.62 ± 0.84 ng/ml, *n* = 6, *p* = 0.0175, two-way ANOVA and Tukey’s multiple comparison) at 2 days, indicating NaB treatment attenuated BBB disruption (Fig. 4a). At 5 days, serum GFAP levels were low, and the two groups did not show significant differences.

### NaB reduces lipid oxidation in brain in the early acute phase

HDAC inhibitors including NaB have shown to attenuate oxidative damage in animal models of bipolar disorder, diabetes, and cancer [28–31]. To assess the effect of NaB on MCAo induced oxidative stress, we measured the levels of thiobarbituric reactive species (TBARS), as a proxy marker of reactive oxygen species [32, 33]. As shown in Fig. 4b, c, at 2 days post stroke TBARS values display a main effect of treatment (NaB) in both hemispheres (Fig. 4b, *p* = 0.0025) whereas at 5 days there was no differences among the treatment groups (Fig. 4c, *p* = 0.2210), indicating that NaB promotes an early protective effect on ischemia-induced oxidative stress in brain.

### NaB-mediated neuroprotection after stroke is associated with the anti-inflammatory effect in circulation and brain

**Fig. 5:**
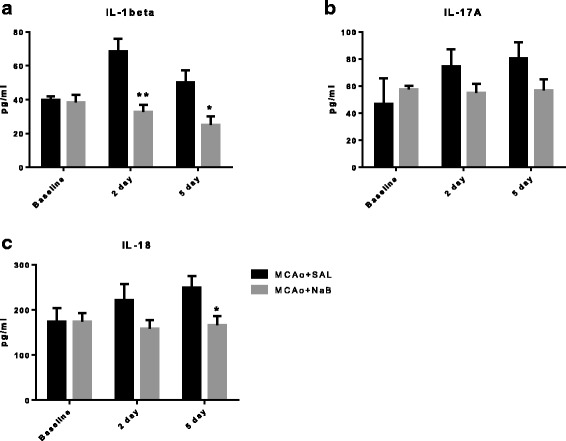
The effect of NaB on inflammatory cytokines in serum. Cytokine levels from serum were evaluated by ELISA on samples obtained at baseline, 2 and 5 days post stroke for IL-1beta (**a**), IL-17A (**b**), and IL-18 (**c**). All *graphs* represent mean ± S.E.M. *n* = 6–9 in each group. **p < 0.01; *p < 0.05; two-way ANOVA with Tukey’s post hoc test

Ischemia induces inflammation, which exacerbates cell death and prolongs recovery from stroke injury. Circulating cytokine levels were examined in serum obtained from the saphenous vein before (baseline) and after (2 and 5 days) stroke. The pro-inflammatory cytokine, IL-1beta, was significantly affected by treatment [F _(1, 30)_, 11.91, *p* = 0.0017]. Serum IL-1beta was significantly elevated in post-stroke saline-treated group at day 2 (68.68 ± 7.19 pg/ml) compared to the levels of baseline (39.76 ± 2.11 pg/ml; *p* = 0.0419), while the NaB-treated group maintained similar levels at baseline (38.45 ± 4.35 pg/ml), 2d (32.88 ± 4.12 pg/ml, *p* = 0.4491 baseline vs. 2 days), and 5 days (25.09 ± 4.72 pg/ml, p = 0.8559 baseline vs. 5 days) post stroke (Fig. 5a). Another pro-inflammatory cytokine, IL-18, was also decreased in post-stroke NaB-treated group (166.2 ± 20.34 pg/ml) as compared to the levels in post-stroke saline-treated group (249.1 ± 25.49 pg/ml) at 5 days (*p* = 0.0366) (Fig. 5c). There was no significant differences in IL-17A levels among the treatment groups (Fig. 5b).

**Fig. 6:**
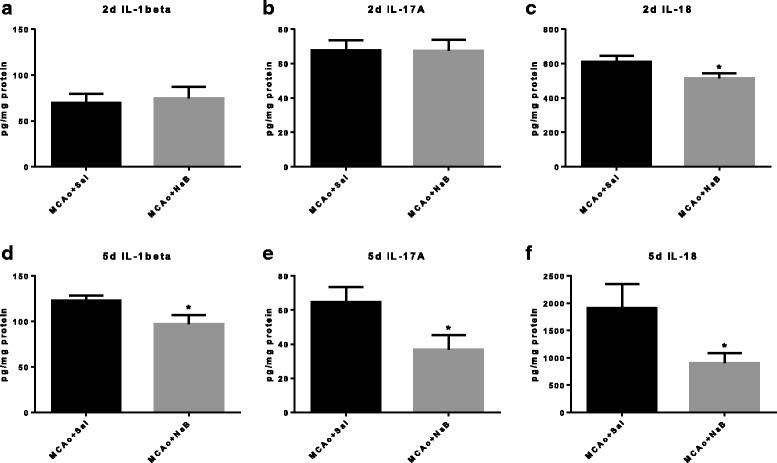
The effect of NaB on inflammatory cytokines in the ischemic hemisphere post stroke. Cytokine levels were measured by ELISA in corticostriatal tissue lysates from the ischemic hemisphere at 2d (**a**–**c**, *n* = 6/group) and 5 days (**d**–**f**, *n* = 10–13/group) post stroke. All *graphs* represent mean ± S.E.M. **p < 0.01; *p < 0.05; unpaired *t* test

Analyses of cytokines in the ischemic hemisphere at 2d post-stroke showed that only one pro-inflammatory cytokine, IL-18, was affected by post-stroke NaB treatment (Fig. 6c, 611.1 ± 34.12 pg/mg protein vs. 515.1 ± 30.23 pg/mg protein, *p* = 0.0467, *n* = 12/group), while IL-1beta (Fig. 6a, 69.86 ± 9.58 pg/mg protein vs. 74.73 ± 12.48 pg/mg protein, *p* = 0.7598, *n* = 12/group) and IL-17A (Fig. 6b, 67.62 ± 6.05 pg/mg protein vs. 67.51 ± 6.31 pg/mg protein, *p* = 0.9902, *n* = 11-12/group) were not affected. At 5 days post stroke, NaB-treatment reduced expression of brain IL-1beta (Fig. 6d, 123.0 ± 5.69 pg/mg protein vs. 97.05 ± 10.19 pg/mg protein, *p* = 0.0419, *n* = 11–12/group), IL-17A (Fig. 6e, 64.55 ± 8.79 pg/mg protein vs. 36.85 ± 8.39 pg/mg protein, *p* = 0.0344, *n* = 11–12/group), and IL-18 (Fig. 6f, 1911 ± 437.3 pg/mg protein vs. 907.8 ± 180.9 pg/mg protein, *p* = 0.0350, *n* = 11–13/group) in the ischemic hemisphere (cortex + striatum).

### The protective effect of NaB against ischemia is associated with elevated levels of insulin-like growth factor-1 (IGF-1)

**Fig. 7:**
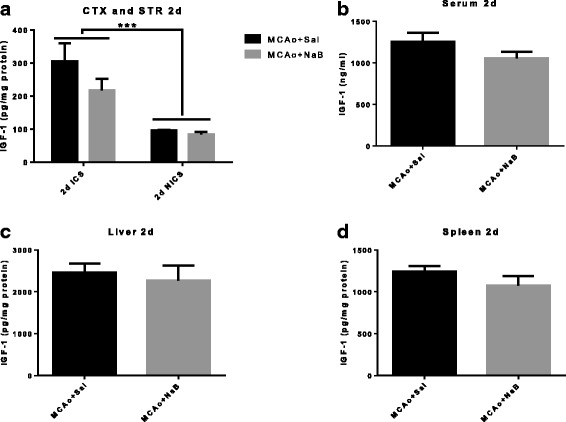
Central and peripheral levels of IGF-1 at 2 days post stroke. IGF-1 levels were measured from cortex and striatum (**a**), serum (**b**), liver (**c**), and spleen (**d**) samples collected at 2 days post MCAo.(a) ***p < 0.001 main effect of hemisphere. Two-way ANOVA. (**b**–**d**) Unpaired *t* test. All *graphs* represent mean ± S.E.M. *n* = 6 in each group. *ICS* ischemic cortex and striatum, *NICS* non-ischemic cortex and striatum

NaB has been shown to regulate several members of the IGF-1 signaling pathway [34, 35]; however, its effect on IGF-1 per se is not well established. Since IGF-1 is a known neuroprotectant for stroke in young and aging animals, we next investigated IGF-1 levels in serum and corticostriatal samples from ischemic and non-ischemic hemispheres Consistent with previous reports [19, 20, 22, 36–38], ET-1 induced MCAo elevated IGF-1 level in ischemic hemisphere in the control (69%) and NaB treated (61%) (Fig. 7a). Ischemia significantly increased brain IGF-1 levels at 2d post stroke, however, brain IGF-1 levels were not affected by NaB treatment [F _(1,10)_, 2.19, *p* = 0.1697] (Fig. 7a). No significant alteration was observed in IGF-1 levels from serum, liver, and spleen between groups at 2 days post stroke (Fig. 7b–d).

**Fig. 8:**
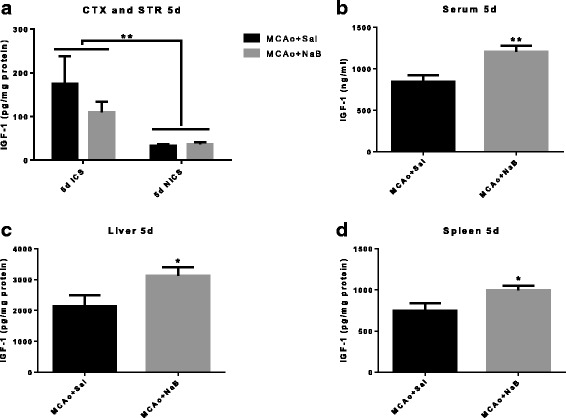
NaB treatment increases IGF-1 levels in peripheral tissues but not brain at 5 days post stroke. IGF-1 levels were determined by ELISA from cortex + striatum (**a**), serum (**b**), liver (**c**), and spleen (**d**) samples collected at 5d post MCAo. **a** IGF-1 levels are significantly elevated in the ischemic hemisphere; however, there is no difference in IGF-1 level between treatment groups in either the ischemic or non-ischemic hemisphere. ***p < 0.001 main effect of hemisphere. **b**–**d** Post-stroke NaB treatment increased serum (**b**), liver (**c**), and spleen (**d**) levels of IGF-1 as compared to post-stroke vehicle-treated group. **p < 0.01; unpaired *t* test. All *graphs* represent mean ± S.E.M. *n* = 6 in each group. *ICS* ischemic cortex and striatum, *NICS* non-ischemic cortex and striatum

Similar to the pattern seen at 2 days post stroke, brain IGF-1 levels at 5 days post stroke were only affected by ischemia (hemisphere) [F _(1,10)_, 10.92, *p* = 0.0079], but not by NaB treatment [F _(1,10)_, 0.74, *p* = 0.4098] (Fig. 8a). Strikingly, 5d IGF-1 analysis showed that post-stroke NaB treatment significantly elevated IGF-1 expression by 28% in serum (1097.89 ± 75.51 vs. 859.66 ± 30.22 ng/ml), by 46% in liver (3123.82 ± 245.99 pg/mg protein vs. 2140.81 ± 345.89 pg/mg protein), and by 34% in spleen (996.86 ± 53.78 vs. 746.12 ± 92.41 pg/mg protein) as compared to post-stroke saline treated group (Fig. 8b–d). Hence, NaB effects on IGF-1 were restricted to peripheral tissues.

### IGF-binding protein-3 (IGFBP-3) is increased in ischemic hemisphere as compared to non-ischemic hemisphere post stroke

**Fig. 9:**
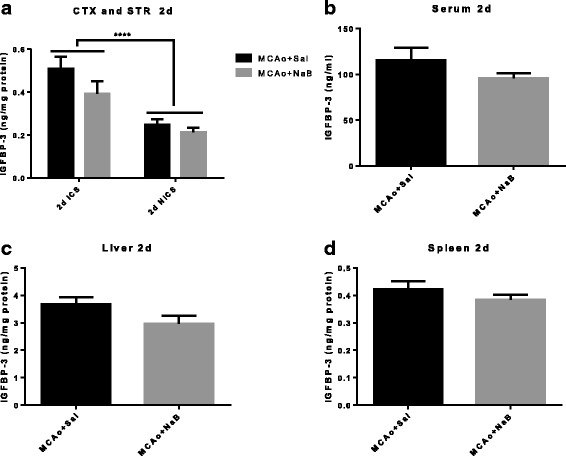
IGFBP-3 level is increased in ischemic hemisphere at 2 days post stroke. IGFBP-3 levels were measured from cortex and striatum (**a**), serum (**b**), liver (**c**), and spleen (**d**) samples collected at 2 days post MCAo. IGFBP3 levels are significantly elevated in the ischemic hemisphere compared to the non-ischemic hemisphere. NaB did not affect IGFBP3 expression in any of these tissue. All *graphs* represent mean ± S.E.M. *n* = 6 in each group. ***p < 0.001 main effect of hemisphere. Two-way ANOVA (**a**) and unpaired *t* test (**b**–**d**). *ICS* ischemic cortex and striatum, *NICS* non-ischemic cortex and striatum

**Fig. 10:**
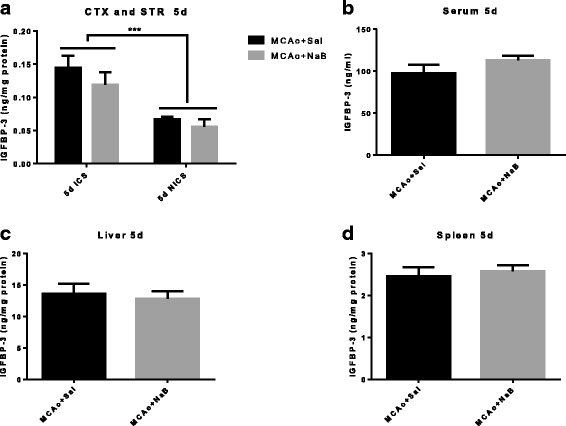
IGFBP-3 level is increased in ischemic hemisphere at 5 days post MCAo. IGFBP-3 levels were measured from cortex and striatum (**a**), serum (**b**), liver (**c**), and spleen (**d**) samples collected at 5 days post stroke. IGFBP3 levels are significantly elevated in the ischemic hemisphere compared to the non-ischemic hemisphere. NaB did not affect IGFBP3 expression in any of these tissue. All *graphs* represent mean ± S.E.M. *n* = 6 in each group. ***p < 0.001 main effect of hemisphere. Two-way ANOVA (**a**) and unpaired *t* test (**b**–**d**). *ICS* ischemic cortex and striatum, *NICS* non-ischemic cortex and striatum

The IGF signaling pathway includes ligands (IGF-1 and -2), receptor (IGF-1R), and binding proteins (IGFBPs) [39]. Since IGFBP-3 is a major circulating IGFBP, which binds >75% of serum IGFs, we next measured IGFBP-3 levels in brain, serum, liver, and spleen at 2d and 5d post stroke. Figures 9a and 10a show that IGFBP-3 is significantly affected by hemisphere, but not by NaB treatment in brain. Similarly, NaB did not affect IGFBP3 expression either at 2 or 5 days post stroke in serum (Figs. 9b and 10b), liver (Figs. 9c and 10c), and spleen (Figs. 9d and 10d).

## Discussion

Our data provide the first evidence that post-stroke NaB treatment is neuroprotective in middle-aged reproductive senescent female rats. In this study, we show that two i.p. injections (6 and 30 h after MCAo) of NaB significantly reduced cortical and striatal infarct volume and ameliorated stroke-induced loss of sensory motor function. Preclinical studies have identified NaB as a potential therapeutic drug for ischemic stroke [11, 40, 41], although these studies have mainly utilized young male animals. While older women have a higher risk for stroke and poorer recovery as compared to aged men, no studies have evaluated the effectiveness of HDAC inhibitors in clinically relevant animal models such as aged animals or females. Furthermore, few studies have treated NaB at a delayed time point (>6 h) following stroke, which is a critical question for stroke therapy [42]. The present study convincingly demonstrates that delayed NaB administration is effective for middle-aged female rats after cerebral ischemia.

While NaB is generally shown to improve stroke outcomes, its mechanism of action appears to be pleiotropic. During the acute phase poststroke, NaB reduces oxidative stress and blood-brain barrier permeability, whereas, during the delayed phase poststroke, NaB promotes cell survival and tissue repair and recovery [43, 44]. At the early phase (ranging from minutes to hours), reactive oxygen species are released from injured cells, further stimulating the release of inflammatory cytokines as well as matrix metalloproteinases, which act in concert to increase blood brain barrier permeability and trafficking of leukocytes [15, 16]. Delayed actions of NaB may contribute to stroke recovery and repair in two ways: one, by elevating IGF-1 in periphery tissues, which may trigger remodeling responses in both endothelial cells and astrocytes [22, 45] and additionally, by decreasing pro-inflammatory cytokines such as IL-17. IL-17 is produced by gammadeltaT lymphocytes, and infiltration of this T cell cohort in the brain during the acute phase of stroke been shown to promote infarction and brain damage [44]. Decreased IL-17 production in NaB group is thus consistent with the reduced infarct volume seen in this group and reducing this inflammatory cytokine may further promote repair processes in delayed phase of stroke.

Unlike its action on lipid peroxides and IGF-1, NaB suppression of inflammatory cytokines spanned the early and late acute phase of stroke. Our data show that NaB decreased pro-inflammatory cytokine levels of IL-1beta in circulation at 2 days post stroke and both IL-18 and IL-1beta at 5 days post stroke. In the ischemic hemisphere, NaB decreased IL-18 at 2 days post stroke and IL-1beta, IL-17a, and IL-18 at 5 days post stroke. Our previous studies show that neither of these proteins are significantly different in young or middle aged females [46], and are likely driven by ischemic injury. Proinflammatory cytokines released during ischemic stroke have a detrimental effect on neuronal survival and functional recovery [47, 48]. Lower levels of IL-18 by NaB at both 2 and 5 days post stroke is particularly interesting in view of the data that this cytokine is reported to have prognostic value in patients with acute ischemic stroke [49–51]. IL-18 is synthesized peripherally by macrophages and human peripheral blood mononuclear cells [52, 53] and by microglia, astrocytes, and neurons in central nervous system as the first response of immune defense [54–56]. Zaremba and colleagues have shown that serum IL-18 level is significantly elevated in stroke patients as compared to controls, and IL-18 levels were negatively correlated with both the Scandinavian Stroke Score and the Barthel Index [50]. Moreover, circulating IL-18 levels from blood drawn 48 h after ischemic stroke was a major predictor of 90-day major adverse clinical outcomes in this cohort [49]. These clinical reports are consistent with our observation in middle-aged female rats where higher IL-18 levels in the saline-treated group is accompanied by worse sensory motor function as compared to the post-stroke NaB-treated group. In addition to the clinical data, preclinical studies using myeloid cells have shown that HDAC inhibitors including NaB, valproic acid and TSA increase expression of IL-18 mRNA [57].

We also found lower levels of IL-17A by NaB in the ischemic hemisphere only at later acute phase of stroke (5 days, Fig. 6e), but not at early acute phase (Fig. 6b), while in peripheral tissues lower levels of IL17A were found both at 2 and 5 days (Fig. 5b). This may imply migration of Th17 cells to the brain at later acute phase of stroke, which is initially activated from periphery at the earlier acute phase. It is plausible because HDAC inhibitors are reported to regulate T cell polarization, by which HDAC inhibition suppresses the polarization toward the pro-inflammatory Th17 cells [58]. Our data also suggest that NaB protect the brain from neuroinflammatory responses, in part, by suppressing peripheral immune responses by reducing trafficking of T-cells.

While the effects of NaB on inflammatory cytokines are extensively studied, its effects on IGF-1 are not well known. The IGF-1 signaling pathway is a critical biological modulator of cellular growth, development, and metabolism [39]. Our laboratory has previously demonstrated that stroke severity in older females is associated with decreased IGF-1 availability [20] and IGF-1 treatment, provided intracerebroventricularly, after stroke protects the aging brain by reducing blood brain barrier disruption and neuroinflammation [22]. Interestingly, while NaB significantly increases IGF-1 in peripheral tissues in the post stroke animal, there was no elevation of IGF-1 in the brain with this treatment. In a study examining the biodistribution of exogenous fatty acids including n-butyric acid (BA), 4-phenylbutyric acid (PBA) and valproic acid in primates [59], only a very small amount (<0.006%) is taken up by the brain, whereas majority of these fatty acids were taken up by spleen (BA) and liver (PBA). Considering this biodistribution, it is not surprising that NaB was most effective in IGF-1 level in liver, spleen, and serum. The relationship between NaB and IGF-1 is poorly understood. NaB has been shown to cause a dose dependent increase in IGFBP3 and IGFBP2 protein and mRNA [34], proteins that are known to store and increase the half-life of IGF-I. Since the dose of NaB used in this study has been reported to restore MCAo-induced decrease in histone acetylation [13], another likely possibility is that increased IGF-1 expression pathway may result from increased transcription due to preservation of histone acetylation.

Stroke-induced inflammation, and the trafficking of immune cells to the ischemic brain is a critical feature of stroke pathophysiology. Young, cycling females typically display small infarct volumes with fewer T cells in the brain after stroke [60], which is positively correlated with serum estradiol levels. Loss of this ovarian hormone in aged females was associated with increased stroke outcome and splenic contraction. In a comprehensive analysis of the central and peripheral immune response to MCAo, Zhang et al. reported that in estrogen-deficient females, stroke increased splenic Treg cells and reduced splenocyte proliferation and thymocyte numbers, while estrogen replacement reduced infarct volume, decreased splenic Treg cell numbers and increased splenic proliferation [61]. These data suggest that the loss of estrogen in middle aged females likely exacerbates the immune cell invasion to the brain and causes worse outcome post stroke. It is also consistent with the idea that NaB may improve infarct volume in this population by modulating the inflammatory response.

While aged women are likely to have a higher risk for stroke, worse outcomes and poorer recovery after the event compared to aged men, preclinical studies routinely failed to utilize clinically relevant animal models, such as aged female model. The present study suggests that readily supplementable and clinically well-tolerated NaB could be an effective treatment to improve sensory motor function and outcome after acute ischemic stroke in middle-aged females, suggesting that HDAC inhibitors might have utility in treating acute stroke in a broad range of demographics. While several studies show that sodium butyrate is neuroprotective, the precise mechanism is unclear, and is likely to be multi-pronged, involving anti-inflammatory, anti-apoptotic and growth factor mediated pathways [9]. The current study shows that NaB may target peripheral organs to promote central nervous system health.

## Conclusions

The results of the present study with a HDAC inhibitor, sodium butyrate (NaB) provide the first evidence that delayed NaB treatment post-stroke is neuroprotective in middle-aged reproductive senescent female rats. Our data indicate that two doses of NaB by i.p. injection (6 and 30 h after MCAo) significantly reduce corticostriatal infarct volume and significantly attenuates loss of sensory motor function. The neuroprotection of NaB against MCAo is associated with an anti-inflammatory effect of this HDAC inhibitor. Additionally, our data show that NaB also an early anti-oxidant effect and a later trophic effect, specifically, elevating IGF-1, which we have previously shown is a robust neuroprotectant for stroke in aging females.

## Abbreviations

BA: n-butyric acid
BBB: Blood-brain barrier
BDNF: Brain-derived neurotrophic factor
COX-2: Cyclooxygenase-2
ET-1: Endothelin-1
GFAP: Glial fibrillary acidic protein
HAT: Histone acetyltransferase
HDAC: Histone deacetylase
IGF-1: Insulin-like growth factor-1
IGFBP3: IGF binding protein 3
IL: Interleukin
Lys: Lysine
MCA: Middle cerebral artery
MCAo: Middle cerebral artery occlusion
NaB: Sodium butyrate
PBA: 4-phenylbutyric acid
TBARS: Thiobarbituric reactive species
TrkB: Tropomyosin receptor kinase B
TTC: 2,3,5-triphenyltetrazolium chloride
